# Conditions under Which Leprosy Has Declined in Iceland

**Published:** 1896-09

**Authors:** 


**Conditions under which Leprosy has Declined in Iceland.** By Edward H. Ehlers, M.D. **Leprosy in South Africa.** By S. P. Impey, M.D. **On Spontaneous Recovery from Leprosy.** By S. P. Impey, M.D. Pp. 79. London: Macmillan & Co. 1895.

These three essays are issued together in one volume, under the auspices of the National Leprosy Fund. The first contains a very interesting account of the history of leprosy in Iceland; but we cannot compliment the Government of the country on the sanitary steps they have taken to deal with the disease. If some intelligent medical officers of health could have their sway there, they could establish a reputation, for even ordinary cleanliness and decent sanitary arrangements seem very much neglected. By the middle of the 16th century leprosy was so extensive in the island, that Gislasen reports that about this period they never mentioned the name of the disease without adding "May God in His mercy save us!" If with this they had combined the next thing to godliness, cleanliness, their prayers might have been answered. It is very difficult to ascertain the number of cases of leprosy in Iceland, because a great many are hidden away; but in 1895 Dr. Ehlers considered they were not fewer than two hundred. It is interesting to notice that three-fourths of the cases are on the south-west coast. It was here that the disease was first imported. This is the part which sustains the commercial communication with other countries, and here the great epidemics of measles, scarlet fever, and small-pox have had their first incidence. Many other parts of Dr. Ehlers's well-written essay are of much interest.

As regards leprosy in South Africa, Dr. Impey shows that until the middle of the last century, leprosy was not prevalent in European settlements; but there is no question that it had existed long before amongst the aborigines, and that the Hottentots (a nomadic race) had spread the disease, as also the Griquas, who were an offshoot from the Hottentots, and that owing to only very indifferent precautions, the disease has been spreading during the last fifty years. From a table (p. 56) of the number of lepers at present known in the various South African colonies and states there would appear to be about 1750, and if only all cases could be segregated like those at Robben Island, about ten miles north-west from Cape Town, where 1,697 have been admitted since 1845, and of which 776 have died (and the various Governments are awakening to the fact that they must do something to check the spread of the disease), Dr. Impey's interesting accounts and statistics will not have been compiled in vain.

Dr. Impey's communication "On Spontaneous Recovery from Leprosy," is an important one. It has been observed from time to time that an attack of measles, small-pox, or erysipelas occurring in a leprous subject may bring about the cure of the leprosy, and as these diseases depend upon a bacillus, the author points out and tries to confirm the idea that the bacilli of these complaints are stronger than the germs of leprosy, and therefore thrust the weaker ones to the wall, and he suggests that the vaccination of lepers with these same bacilli may effect cures of leprosy. It may be that the incidence of these complaints uses up the pabulum that the bacillus lepræ thrives upon. Anæsthetic leprosy would seem, however, in some cases to work out its own cure in time, and the author gives some valuable evidence to show that patients who live for more than eleven years after having been attacked with this form are practically cured, although they may bear the marks, so to say, of the disease—just as small-pox patients may display severe brands of their attack, but yet are not suffering from small-pox. Thus perforating ulcer very frequently is kept up, not by leprosy, but by the bare bone beneath being diseased. Remove this latter and the ulcer heals. In these cases (self-cured) when the anæsthetic parts are accidentally injured, as must occur occasionally, these wounds do not show any leprous taint; they heal well, and tissue-sections display no bacilli.

